# Toll-like receptor 2 downregulation and cytokine dysregulation predict mortality in patients with *Staphylococcus aureus* bacteremia

**DOI:** 10.1186/s12879-020-05641-z

**Published:** 2020-11-30

**Authors:** Nak-Hyun Kim, Ji Yeon Sung, Yoon Jung Choi, Su-Jin Choi, Soyeon Ahn, Eunjeong Ji, Moonsuk Kim, Chung Jong Kim, Kyoung-Ho Song, Pyoeng Gyun Choe, Wan Beom Park, Eu Suk Kim, Kyoung Un Park, Nam-Joong Kim, Myoung-don Oh, Hong Bin Kim

**Affiliations:** 1 Department of Internal Medicine, Seoul National University Hospital, Seoul National University College of Medicine, Seoul, Republic of Korea; 2 Department of Internal Medicine, Seoul National University Bundang Hospital, Seoul National University College of Medicine, 173 Gumi-ro, Bundang-gu, Seongnam, 463-707 Republic of Korea; 3 Department of Laboratory Medicine, Seoul National University Bundang Hospital, Seoul National University College of Medicine, Seongnam, Republic of Korea; 4 Present Address: Roche Korea, Seoul, Republic of Korea; 5 Medical Research Collaborating Center, Seoul National University Bundang Hospital, Seongnam, Republic of Korea; 6 Present Address: Department of Internal Medicine, Ewha Womans University, Seoul Hospital, Seoul, Republic of Korea

**Keywords:** *Staphylococcus aureus*, Bacteremia, Toll-like receptor 2 (TLR2), Cytokines

## Abstract

**Background:**

*Staphylococcus aureus* bacteremia (SAB) presents heterogeneously, owing to the differences in underlying host conditions and immune responses. Although Toll-like receptor 2 (TLR2) is important in recognizing *S. aureus*, its function during *S. aureus* infection remains controversial. We aimed to examine the association of TLR2 expression and associated cytokine responses with clinical SAB outcomes.

**Methods:**

Patients from a prospective SAB cohort at two tertiary-care medical centers were enrolled. Blood was sampled at several timepoints (≤5 d, 6–9 d, 10–13 d, 14–19 d, and ≥ 20 d) after SAB onset. TLR2 mRNA levels were determined via real-time PCR and serum tumor necrosis factor [TNF]-α, interleukin [IL]-6, and IL-10 levels were analyzed with multiplex-high-sensitivity electrochemiluminescent ELISA.

**Results:**

TLR2 levels varied among 59 SAB patients. On days 2–5, TLR2 levels were significantly higher in SAB survivors than in healthy controls (*p* = 0.040) and slightly but not significantly higher than non-survivors (*p* = 0.120), and SAB patients dying within 7 d had lower TLR2 levels than survivors (*P* = 0.077) although statistically insignificant. IL-6 and IL-10 levels were significantly higher in non-survivors than in survivors on days 2–5 post-bacteremia (*P* = 0.010 and *P* = 0.021, respectively), and those dying within 7 d of SAB (*n* = 3) displayed significantly higher IL-10/TNF-α ratios than the survivors did (*P* = 0.007).

**Conclusion:**

TLR2 downregulation and IL-6 and IL-10 concentrations suggestive of immune dysregulation during early bacteremia may be associated with mortality from SAB. TLR2 expression levels and associated cytokine reactions during early-phase SAB may be potential prognostic factors in SAB, although larger studies are warranted.

**Supplementary Information:**

The online version contains supplementary material available at 10.1186/s12879-020-05641-z.

## Background

*Staphylococcus aureus* is an opportunistic pathogen causing various pathological conditions, ranging from asymptomatic colonization to virulent invasive infections including bacteremia [1]. The clinical presentation of *S. aureus* bacteremia (SAB) is heterogeneous; while many patients experience only uncomplicated bacteremia, others exhibit metastatic infections in various organ systems that warrant prolonged antibiotic therapy and often lead to a poor prognosis due to serious sequelae [2]. Many risk factors affecting prognosis in SAB have been identified; host factors include old age [3, 4], presence of comorbidities [5], source of infection and severity of infection at time of presentation [6], and underlying immune status [7, 8], while pathogen-specific factors include methicillin- resistance [9], exotoxins produced by *S. aureus*, and specific *S. aureus* strains [10, 11]. Other than virulence factors and antibiotic susceptibility of the infecting *S. aureus*, underlying host conditions, and host immune responses contribute to case-by-case variation in host-pathogen relationships, thus resulting in clinical heterogeneity [12–14]. Nevertheless, complex mechanisms underlying the host responses remain unclear.

Upon bloodstream invasion, *S. aureus* is initially recognized and cleared from circulation by the innate immune system, which is initiated through a sequence of events resulting in the production and secretion of various inflammatory cytokines and chemokines, phagocyte activation, and the initiation of adaptive immunity [14]. Toll-like receptors (TLRs), which are pattern recognition receptors (PRRs) on phagocytic cell membranes, play a pivotal role in recognizing pathogen-associated molecular patterns on bacterial cell walls and activating intracellular signaling pathways [15]. In particular, TLR2 has been reported to be involved in the recognition of staphylococcal peptidoglycan, lipoteichoic acid, and lipoproteins [14, 16, 17]. Upon activation, TLR2 triggers inflammatory signaling pathways, resulting in increased expression and secretion of various antimicrobial peptides, cytokines, and chemokines, which recruit immune cells to the site of infection and trigger the adaptive immune response [17–19]. Notably, *S. aureus* produces staphylococcal superantigen-like protein 3 (SSL3) and TIR-containing protein (TirS) during infections, which interfere with TLR2 signaling and dampen appropriate TLR2 activation [20–23].

Although TLR2-mediated immune responses during staphylococcal infections have been extensively studied, most evidence has been obtained from in vitro studies or in vivo murine models, with varying outcomes [17, 21]. While immune responses primarily triggered through TLR2 signaling are pro-inflammatory, immunoregulatory responses mediated by TLR2 have also been reported [16, 24]. Some human studies on TLR2 expression in sepsis patients have reported that TLR2 is upregulated in sepsis patients and down-regulated in severe sepsis and septic shock patients, leading to death [25–27]. However, studies on SAB in humans are scarce [27, 28].

Therefore, the aims of this study were to assess TLR2 expression and associated host cytokine responses during the course of SAB, and to examine how they differ on the basis of clinical outcomes of SAB at different stages of the infection.

## Methods

### Patients

SAB patients older than 18 years were enrolled from a prospective SAB cohort from March 2014 to April 2015 in two tertiary-care hospitals in Korea: Seoul National University Hospital, Seoul and Seoul National University Bundang Hospital, Gyeonggi-do. Cases of suspected contamination and polymicrobial infection were excluded, along with patients with a WBC count less than 4000/μL, and those who declined to participate in the study.

Clinical data including demographic characteristics, Charlson’s comorbidity-weighted index (CCWI) score [29], severity of acute infection as measured on the basis of the Pitt bacteremia score [30] and sequential organ failure assessment (SOFA) score [31], nosocomial or community-acquired bacteremia with a history of previous health care contact [32], antimicrobial therapy, in-hospital mortality, and methicillin resistance of blood isolates were obtained. Appropriate antimicrobial therapy was grossly defined as treatment with susceptible antibiotics based on antimicrobial susceptibility test (AST) results; for methicillin-resistant *S. aureus* (MRSA), appropriate antimicrobial therapy included glycopeptides (with therapeutic drug monitoring for vancomycin), linezolid, and alternative agents according to AST results when treatment with glycopeptides or linezolid was not feasible, and for methicillin-susceptible *S. aureus* (MSSA), β-lactams and alternative agents based on AST results, in case of β-lactam hypersensitivity, were regarded appropriate [2]. SAB-related mortality was defined as death within 30 d of SAB onset without other apparent causes of death [33]. Persistent bacteremia was defined as the isolation of *S. aureus* from blood cultures on > 4 consecutive days despite treatment with appropriate antibiotics [34, 35].

### Sample collection

We used timed collected residual peripheral blood samples to analyze TLR2 expression levels and cytokine levels. The samples were obtained at five different timepoints: within 5 d (≤D5), 6–9 d (D6–9), 10–13 d (D10–13), 14–19 d (D14–19), and after 20 d (≥D20) of bacteremia onset. Day 0 was defined as the day of the first *S. aureus*-positive blood culture. Among the individuals discharged (transferred) or those who died within 14 d of admission, only one or two samples were obtained. If more than one sample was available within one period, average TLR2 expression levels were obtained for the samples in that period. Twenty-five healthy volunteers [22 females and 3 males; mean age, 32 years (range 22–46 years)], free of signs of inflammation and underlying diseases, were included in the control group.

### Ethical approval

This study was approved by the Committee of Institutional Ethics Review Board at Seoul National University Hospital (1403–024-562) and Seoul National University Bundang Hospital (B-1402-237-002). All participants, including healthy volunteers, provided written informed consent unless the requirement for informed consent was waived.

### Measurement of TLR2 expression

RNA was extracted from whole blood using QIAamp RNA Blood Mini kit (Qiagen, Valencia, CA, USA) in accordance with the manufacturer’s instructions. Extracted RNA was either directly converted to cDNA through RT-PCR or frozen at − 70 °C. RT-PCR was performed using SuperScript III Reverse Transcriptase (Invitrogen, Carlsbad, CA, USA). cDNA was stored at − 70 °C until amplified through real-time quantitative PCR (qPCR). Real-time qPCR was performed to measure *TLR2* expression levels using TaqMan Gene Expression Assays (Life Technologies, Carlsbad, CA, USA) for *TLR2* (cat# 4331182, Hs00610101_m1) and *GAPDH* (cat# 4448484, Hs99999905_m1). mRNA levels were quantified using an ABI7500 system (Applied Biosystems, Foster City, CA, USA). *TLR2* mRNA expression levels were determined as the ratio of *TLR2* mRNA to *GAPDH* mRNA using the 2^-ΔΔCt^ method [36]. Each sample was measured in triplicate and the mean value was used in the calculations. We used the MIQE guideline as a reference [37].

### Measurement of cytokine concentrations

Serum was separated from whole blood and frozen at − 70 °C for future use. The concentration of three cytokines, tumor necrosis factor-alpha (TNF-α), interleukin-6 (IL-6), and IL-10 were measured in duplicate, using a V-PLEX customized panel kit (Meso Scale Discovery [MSD], Rockville, MD, USA) in accordance with the manufacturer’s protocol. Data were acquired using a SECTOR S 600 plate reader (MSD, Rockville, MD, USA).

### Statistical analysis

Statistical analyses were primarily performed using IBM SPSS statistics (version 22.0 software package; SPSS Inc., Chicago, IL, USA) and plotted using GraphPad Prism (version 6.01; GraphPad Software, San Diego, CA, USA). Linear mixed modelling was conducted using R 2.10 (R Foundation, Vienna, Austria). Data are presented as mean ± standard deviation (SD), median ± interquartile range (IQR), and proportions, depending on data distribution type. To compare baseline demographics and clinical data, χ^2^ tests and Fisher’s exact tests were performed for categorical variables and independent *t*-tests, and Mann-Whitney U-tests were performed for continuous variables, respectively.

Longitudinal analyses of TLR2 expression and cytokine levels were conducted through linear mixed modelling (LMM), adjusting for sex, age and 30-day mortality, severity of infection, methicillin resistance of *S. aureus* blood isolates, and complicated bacteremia. A two-tailed *p* < 0.05 was considered statistically significant.

## Results

### Clinical and microbiological characteristics

**Fig. 1:**
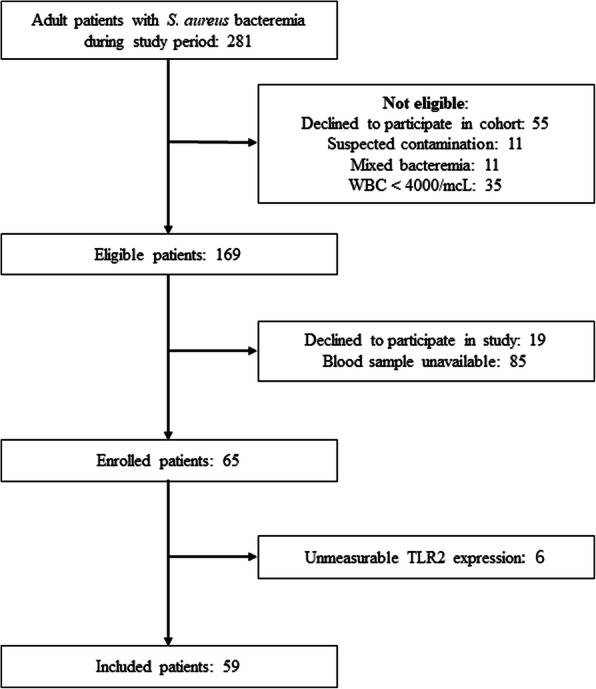
Schematic representation of the protocol for patient selection

Among 281 SAB patients, 109 were considered eligible and 65 patients consented to participate. Real-time PCR results were not obtained for six patients, thus yielding 59 patients for analysis (Fig. 1). Age of patients ranged from 26 to 85 years (mean ± standard deviation, 60.7 ± 16.3) and 78.0% (46/59) were male. MRSA was isolated in 61.0% (36/59). SAB-related mortality was 16.9% (10/59), and in-hospital mortality was 18.6% (11/59). The most frequent primary focus of bacteremia was from a central venous catheter (25.4%, 15/59). The clinical characteristics of the 59 patients enrolled are summarized in Tables 1 and 2, and a comparison of the clinical characteristics of the enrolled and unenrolled patients in the SAB cohort is summarized in Supplementary Table 1.

**Table 1:** Clinical characteristics of patients (*n* = 59)

Characteristics	N (%)^a^
Age (mean [range]) (years)	60.7 [26–85]
Male	46 (78.0)
MRSAB	36 (61.0%)
Length of total hospital stay (median [IQR]) (d)	27.0 [17.0–46.0]
Duration of bacteremia (median [IQR]) (d)	2.0 [1.0–4.0]
Onset of infection	
Community-associated	11 (18.6)
Community-onset, healthcare-associated	22 (37.3)
Hospital-onset	26 (44.1)
In ICU at first positive blood culture	5 (8.5)
CCWI score (median [IQR])	5.0 [2.0–7.0]
Pitt bacteremia score (median [IQR])	1.0 [0.0–3.0]
SOFA score (median [IQR])	4.0 [1.0–8.0]
Severity	
Non-sepsis	9 (15.3)
Sepsis	35 (59.3)
Severe sepsis	8 (13.6)
Septic shock	7 (11.9)
Primary site of infection	
Central venous catheter	15 (25.4)
Bone and joint	14 (23.7)
Skin and soft tissue	7 (11.9)
Lower respiratory tract	6 (10.2)
Cardiovascular site^b^	9 (15.3)
Unknown	6 (10.2)
Others^c^	2 (3.4)
Treatment	
Appropriate empirical	40 (67.8)
Appropriate definitive^d^	59 (100)
Time until appropriate antibiotic (mean ± SD) (h)	30.5 ± 26.3
SAB-related 30-day mortality	10 (16.9)
Persistent SAB	14 (23.7%)
Metastatic SAB	6 (10.2)

^a^ unless otherwise specified

^b^ includes infective endocarditis (3) and other endovascular infections (6)

^c^ intraabdominal (1), and urinary tract infection (1)

^d^ treatment with susceptible antibiotics

*SD* standard deviation, *MRSAB* methicillin-resistant *S. aureus* bacteremia, *IQR* interquartile range, *CCWI* Charlson’s comorbidity-weighted index, *SOFA* sequential organ failure assessment

**Table 2:** Comparison of clinical characteristics of *Staphylococcus aureus* bacteremia patients based on 30-day mortality

Characteristics	Survivors (*n* = 49)^a^	Non-survivors (*n* = 10)^a^	*P*
Age (mean [range]) (years)	59.4 [26–85]	66.8 [48–85]	0.166
Male	38 (77.6)	8 (80.0)	0.865
MRSAB	29 (59.2%)	7 (70.0)	0.523
Length of total hospital stay (median [IQR]) (d)	28.0 [20.0–49.0]	14.5 [7.5–35.3]	0.022
Duration of bacteremia (median [IQR]) (d)	1.0 [1.0–3.5]	4.5 [1.0–5.3]	0.272
Onset of infection			0.592
Community-associated	10 (20.4)	1 (10.0)	
Community-onset, healthcare-associated	17 (34.7)	5 (50.0)	
Hospital-onset	22 (44.9)	4 (40.0)	
Location at the time of first positive blood culture			0.338
General ward	20 (40.8)	4 (40.0)	
ICU	3 (6.1)	2 (20.0)	
Emergency room	26 (53.1)	4 (40.0)	
CCWI score (median [IQR])	4.0 [1.5–7.0]	6.0 [3.0–8.0]	0.174
Pitt bacteremia score (median [IQR])	1.0 [0.0–2.0]	2.0 [1.0–4.0]	0.032
SOFA score (median [IQR])	4.0 [1.0–7.0]	7.0 [5.3–9.8]	0.042
Severity			0.577
Non-sepsis	7 (14.3)	2 (20.0)	
Sepsis	31 (63.3)	4 (40.0)	
Severe sepsis	6 (12.2)	2 (20.0)	
Septic shock	5 (10.2)	2 (20.0)	
Primary site of infection			0.770
Central venous catheter	12 (24.5)	3 (30.0)	
Bone and joint	13 (26.5)	1 (10.0)	
Skin and soft tissue	5 (10.2)	2 (20.0)	
Lower respiratory tract	4 (8.2)	2 (20.0)	
Cardiovascular site^b^	8 (16.3)	1 (10.0)	
Unknown	5 (10.2)	1 (10.0)	
Others^c^	2 (4.1)	0 (0.0)	
Treatment			
Appropriate empirical	35 (71.4)	5 (50.0)	0.266
Appropriate definitive^d^	49 (100)	10 (100)	
MRSA			
Vancomycin (n)	34	7	
Linezolid (n)	1		
Fluoroquinolone (n)	1		
MSSA			
Nafcillin (n)	16	3	
Cefazolin (n)	3		
Ampicillin (n)	1		
Time until appropriate antibiotic (mean ± SD) (h)	31.0 ± 28.0	28.0 ± 16.4	0.911
Persistent SAB	9 (18.4)	5 (50.0)	0.047
Metastatic SAB	4 (8.2)	2 (20.0)	0.266

^a^Data are given as number (%), unless otherwise specified

^b^ includes infective endocarditis and other endovascular infections

^c^ surgical wound, and urinary tract infection

^d^ treatment with susceptible antibiotics

*MRSAB* methicillin-resistant *S. aureus* bacteremia, *IQR* interquartile range, *CCWI* Charlson’s comorbidity-weighted index, *SOFA* sequential organ failure assessment, *MSSA* methicillin-susceptible *S. aureus*

### *TLR2* mRNA expression in SAB patients and healthy controls

Patient blood samples were collected at ≤D5, D6–9, D10–13, D14–19, and ≥ D20 (range: D2–48) post-bacteremia. Temporal changes in *TLR2* mRNA levels varied among patients: *TLR2* mRNA was upregulated in some patients and downregulated in others (Supplementary Fig. 1).

**Fig. 2:**
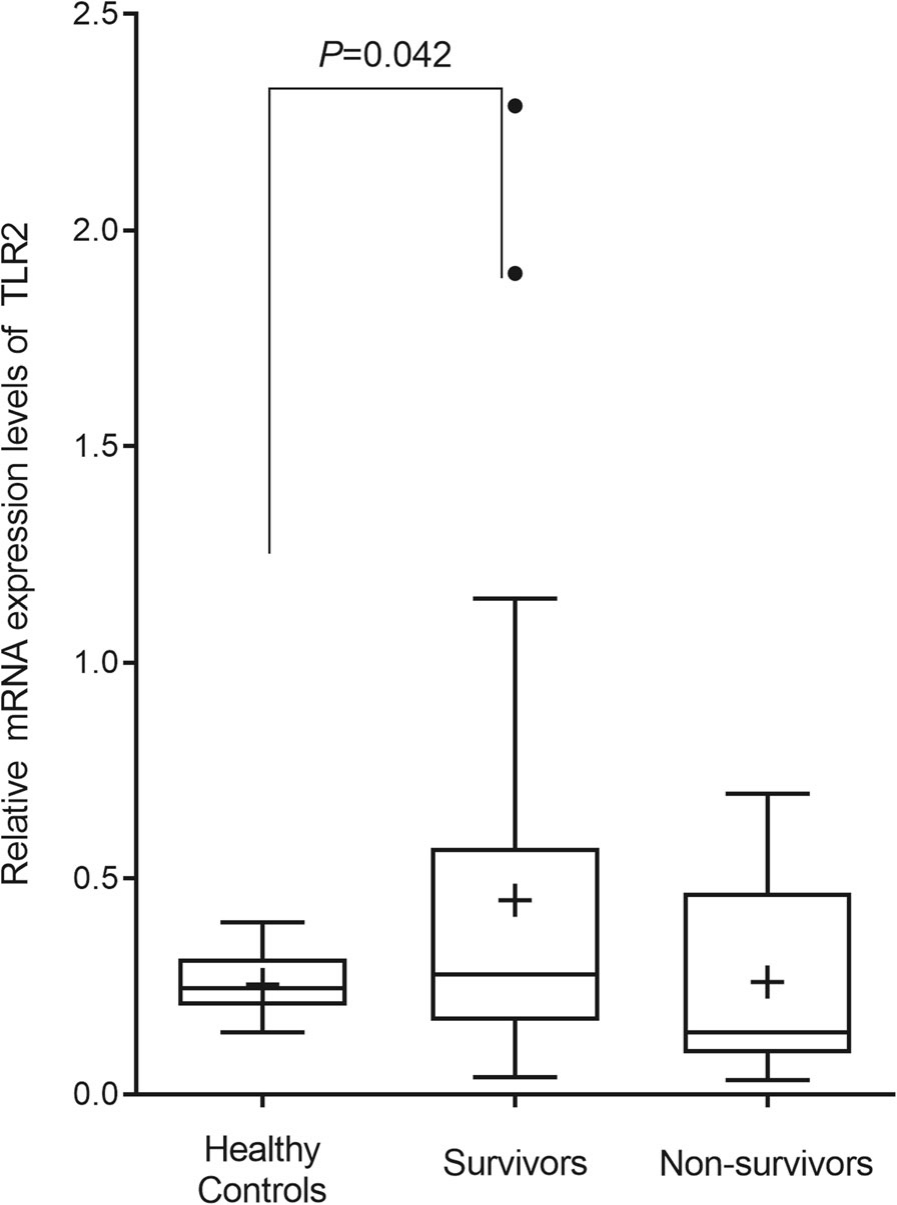
Relative *TLR2* mRNA expression levels within day 5 of *Staphylococcus aureus* bacteremia based on 30-d mortality in comparison with healthy volunteers

Furthermore, during ≤D5 post-bacteremia, TLR2 mRNA expression levels were significantly higher among SAB survivors than among healthy controls (*p* = 0.040) and slightly but not significantly higher than those among patients who died within 30 d of onset of bacteremia, with less dynamic changes (*p* = 0.120) (Fig. 2; Supplementary Fig. 2).

### Host cytokine concentrations and *TLR2* expression in SAB and their association with severity and clinical outcomes

Longitudinal analyses by LMM revealed that TNF-α, IL-6, and IL-10 concentrations were significantly increased in SAB patients who died within 30 d post-bacteremia (with SAB patients who survived as reference) (*p* = 0.001, *p* = 0.002, and *p* < 0.001, respectively). Furthermore, IL-6 and IL-10 levels were significantly increased in patients with septic shock (relative to individuals without sepsis as controls) (*p* = 0.010, and *p* = 0.033, respectively); IL-6 levels were significantly increased in patients who developed metastatic infections (relative to individuals without metastatic infections as controls) (*p* = 0.016).

**Fig. 3:**
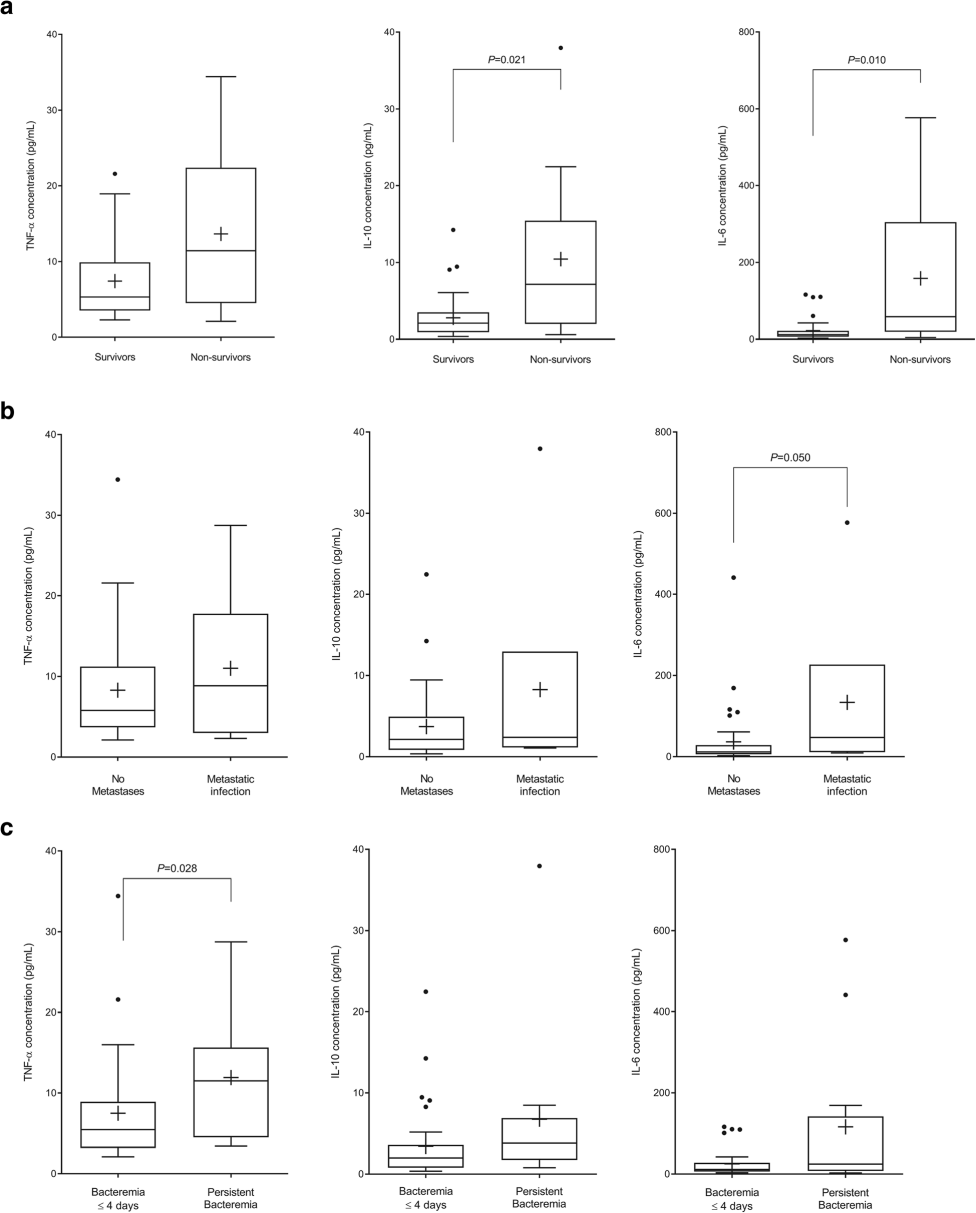
Cytokine concentrations within day 5 of *Staphylococcus aureus* bacteremia based on (**a**) 30-d mortality, (**b**) development of metastatic infections, and (**c**) persistence of bacteremia. **a** 30-day mortality. **b** development of metastatic infections. **c** persistence of bacteremia

When compared within each time period, during ≤D5 post-bacteremia, IL-6 and IL-10 levels were significantly higher in SAB patients who died within 30 d (median [IQR], 58.43 [19.12–305.07] vs 11.69 [6.19–21.51] pg/mL, *p* = 0.010 and 7.15 [1.99–15.46] vs 2.08 [0.90–3.50] pg/mL, *p* = 0.021, respectively) than in the SAB survivors. Furthermore, IL-6 and TLR2 levels were significantly higher in patients who developed metastatic infections than in those who did not develop metastatic infections (IL-6: 46.69 [11.24–226.78] vs 11.80 [6.14–24.48] pg/mL, *p* = 0.050; TLR2: 0.63 [0.29–1.29] vs 0.24 [0.13–1.44], *p* = 0.046). Moreover, TNF-α levels were significantly higher in patients with persistent bacteremia than in those with short-term bacteremia (11.51 [4.51–15.65] vs 5.46 [3.18–8.91] pg/mL, *p* = 0.028) (Fig. 3). IL-6 and IL-10 levels and the IL-10/TNF-α ratio were higher and TLR2 expression levels were lower in SAB patients who died within 7 d (*n* = 3) than in SAB survivors (Supplementary Table 2). During D10–13 post-bacteremia, TNF-α, IL-10, and IL-6 levels were significantly higher in patients with persistent bacteremia than in patients whose SAB resolved in 4 d (TNF-α: 10.41 [4.23–17.14] vs 4.36 [3.31–9.39] pg/mL, *p* = 0.038; IL-10: 2.69 [1.71–4.92] vs 1.18 [0.68–2.10] pg/mL, *p* = 0.038; IL-6: 17.54 [6.58–31.68] vs 6.49 [2.85–12.62] pg/mL, *p* = 0.018). Cytokine profiles and TLR2 expression levels among the outcome groups did not display significant differences at all other timepoints.

## Discussion

Host immune responses during infection are one of the factors contributing to the clinical outcomes of SAB, and previous studies have reported that the marked heterogeneity in the clinical spectrum of SAB may result from differential inflammatory responses to *S. aureus* [12, 13, 34, 38]. TLR2 is reportedly the key sensor of *S. aureus* infections and the primary trigger for innate immune responses [17]. However, available literature contains diverse descriptions of TLR2 function in the immune response to *S. aureus* infections [16, 17, 24], and evidence from human studies are limited. TLR2 expression and associated cytokine levels were analyzed during the course of SAB to investigate their associations with SAB severity and clinical outcomes. The present data show that TLR2 is expressed during early stage SAB, and TLR2 downregulation may be associated with mortality in SAB. Furthermore, the levels of pro- and anti-inflammatory cytokines were significantly higher in SAB non-survivors than in survivors, suggesting the dysregulation of inflammatory responses.

Previous murine studies have reported that TLR2-deficient mice are more susceptible to *S. aureus* infections with a higher bacterial burden and resulted in a higher mortality rate [15]. TLR2 deficiency is suggested to impair phagocytosis, attenuate pro-inflammatory cytokine production, and lead to high mortality [15, 39]. Similarly, in a study on sepsis patients, TLR2 was upregulated in sepsis; however, mortality was associated with TLR2 downregulation [25]. Our results, although not statistically significant, are consistent with these findings, suggesting that TLR2-activity triggered upon bacteremia onset is important for early bacterial clearance and more favorable clinical outcomes of SAB. The lack of statistical significance could be explained by the complex interaction between TLR2 and *S. aureus* in vivo. First, TLR2-activity seems to be influenced by multiple bacterial factors including proliferative activity, capsule formation, protein synthesis, and cell-wall active factors. Hilmi et al. reported marked variability in TLR activity toward *S. aureus* isolates, displaying low to absent TLR2-activity in 64% (68/106) of the isolates tested [40]. Furthermore, Hanzelmann et al. reported strain-specific TLR2-activity among *S. aureus* isolates, showing that strong TLR2 stimulation depends on an active AGR system and is associated with high level phenol-soluble modulin (PSM) production [41]. Second, the route and site of infection seem to influence TLR2-mediated immune responses, as reported in murine studies using TLR2-deficient mice [21]. Although protective roles of TLR2 signaling have been suggested in models of intravenous infection, localized responses to infections have displayed variable outcomes [15, 21]. Third, antimicrobial therapy is also likely to affect the expression of PRRs, including TLR2, by various mechanisms. Testro et al. reported that TLR4 expression was upregulated by antibiotic prophylaxis targeting Gram-negative bacteria in patients with liver cirrhosis [42], and Moore et al. showed that beta-lactams enhanced TLR2 activation by inducing structural changes on the surface of pneumococci, whereas vancomycin did not [43]. Using an in vitro sepsis model, Bode et al. examined the immunomodulatory effects of quinolones, tetracyclines, and macrolides and showed that TLR expression on monocytes and peripheral blood mononuclear cells was differently modulated according to antibiotic treatment [44]. Fourth, host responses to *S. aureus* infections are complex and other PRRs, including NOD-like receptors and C-type lectin receptors, are also involved in bacterial recognition and the induction of inflammatory reactions in response to *S. aureus* infections [22]. The complexity of real-life circumstances dampens the significance of TLR2-activity under controlled in vitro conditions.

Both pro- and anti-inflammatory responses occur during *S. aureus*-induced TLR2 activation [15, 16], along with dysregulated cytokine responses, characterized by elevated IL-10 levels and IL-10/TNF ratios, which are considered responsible for the variations in the clinical outcomes of SAB [12, 13, 38]. Previous studies involving sepsis patients have reported that elevated IL-6 levels are associated with sepsis severity and death [45], and McNicholas et al. reported that IL-6 levels are significantly increased in patients with complicated SAB [12]. Elevated IL-10 levels and a high IL-10/TNF-α ratio have been associated with mortality in severe sepsis patients [46], and similar findings have been previously reported among SAB patients [38]. Consistent with the literature, in this study, patients who died from SAB had significantly higher IL-6 and IL-10 levels during early-stage bacteremia, and patients who died within 7 days of SAB onset further displayed a significantly elevated IL-10/TNF-α ratio and TLR2 downregulation. These results suggest that dysregulated immune responses contribute to SAB-related mortality.

This is the first study to investigate the association among TLR2 expression, cytokine responses, and clinical outcomes in SAB patients. We measured TLR2 expression and cytokine levels throughout the course of SAB (range D2–48) to examine their association with SAB severity and clinical outcomes. Although a correlation between cytokine levels and TLR2 expression could not be determined, our results indicate TLR2 downregulation and significant immune dysregulation in SAB patients with early mortality during early-stage SAB. Furthermore, TNF-α, IL-6, and IL-10 levels were elevated in persistent SAB patients on D10–13.

This study has some limitations, particularly as this study was performed under actual clinical conditions. First, this study was part of a prospective cohort study, not an interventional study; hence, clinical samples could not be obtained at the same time points post-bacteremia, i.e., the timing and total numbers of post-bacteremia blood samples from each patient was different. Therefore, for statistical analysis, we arbitrarily assigned sampling time points as ≤D5, D6–9, D10–13, D14–19 and ≥ D20, thus potentially resulting in bias. However, as in most patients, since the first sample was collected at D3–5, we believe that the measurements during early-stage SAB are largely comparable. Second, since we only included patients who consented to participate, clinically unstable patients were less likely to be enrolled, and the number of patients with worse outcomes was relatively small. Although SAB mortality was lower than that reported in previous studies [47], the clinical characteristics of the enrolled patients and the excluded patients did not significantly differ. Third, we did not analyze bacterial factors including the AGR status, toxigenicity, or inoculum doses, which could have influenced the immune responses and clinical outcomes.

## Conclusions

This study indicated that TLR2 downregulation, and IL-6 and IL-10 elevation during early SAB may be associated with mortality from SAB. Furthermore, dysregulated inflammatory responses characterized by elevated IL-10/TNF-α ratios were associated with mortality within 7 d of SAB onset. Therefore, TLR2 expression levels and associated cytokine reactions during the early-stage may be considered potential prognostic factors in SAB.

## Supplementary Information

**Additional file 1: Supplementary Table 1.** Comparison of clinical characteristics of enrolled and unenrolled patients with *Staphylococcus aureus* bacteremia from March 2014 to April 2015. **Supplementary Table 2.** Relative *TLR2* mRNA expression levels and cytokine concentrations within day 5 of *Staphylococcus aureus* bacteremia (SAB) based on early mortality (mortality within 7 d of SAB onset). **Supplementary Fig. 1.** Relative *TLR2* mRNA expression levels relative to those of *GAPDH* mRNA among all patients. **Supplementary Fig. 2.** Relative *TLR2* mRNA expression levels based on 30-d mortality.

## Data Availability

The datasets generated and analysed during the current study are available from the corresponding author on reasonable request.

## Abbreviations

SAB: *Staphylococcus aureus* bacteremia
TLR: Toll-like receptor
PRRs: Pattern recognition receptors
SSL3: Staphylococcal superantigen-like protein 3
TirS: TIR-containing protein
CCWI: Charlson’s comorbidity-weighted index
SOFA: Sequential organ failure assessment
AST: Antimicrobial susceptibility test
MRSA: Methicillin-resistant *S. aureus*
MSSA: Methicillin-susceptible *S. aureus*
qPCR: Quantitative PCR
TNF: Tumor necrosis factor
IL: Interleukin
SD: Standard deviation
IQR: Interquartile range
LMM: Linear mixed modelling
PSM: Phenol-soluble modulin
