# Supplementary material for: Toll-like receptor 2 downregulation and cytokine dysregulation predict mortality in patients with Staphylococcus aureus bacteremia
Source: BMC Infect Dis. 2020 Nov 30;20:901. doi: 10.1186/s12879-020-05641-z (PMC7706030; doi:10.1186/s12879-020-05641-z)
Supplement: Supplementary file 1 — Additional file 1: Supplementary Table 1. Comparison of clinical characteristics of enrolled and unenrolled patients with Staphylococcus aureus bacteremia from March 2014 to April 2015. Supplementary Table 2. Relative TLR2 mRNA expression levels and cytokine concentrations within day 5 of Staphylococcus aureus bacteremia (SAB) based on early mortality (mortality within 7 d of SAB onset). Supplementary Fig. 1. Relative TLR2 mRNA expression levels relative to those of GAPDH mRNA among all patients. Supplementary Fig. 2. Relative TLR2 mRNA expression levels based on 30-d mortality. [file 12879_2020_5641_MOESM1_ESM.docx]

Supplementary Table 1. Comparison of clinical characteristics of enrolled and unenrolled patients with *Staphylococcus aureus* bacteremia from March 2014 to April 2015

| Characteristics | Enrolled (n=59) | Unenrolled (n=110) | *P* |
| --- | --- | --- | --- |
| Age (mean [range]) (years) | 60.7 [26-85] | 64.4 [19-88] | 0.140 |
| Male | 46 (78.0) | 61 (55.5) | 0.004 |
| MRSAB | 36 (61.0%) | 57 (51.8) | 0.252 |
| Length of total hospital stay (median [IQR]) (d) | 27.0 [17.0-46.0] | 19.0 [5.0-35.0] | 0.615 |
| Duration of bacteremia (median [IQR]) (d) | 2.0 [1.0-4.0] | 1.0 [1.0-3.0] | 0.112 |
| Onset of infection |  |  | 0.974 |
| Community-associated | 11 (18.6) | 22 (20.0) |  |
| Community-onset, healthcare-associated | 22 (37.3) | 41 (37.3) |  |
| Hospital-onset | 26 (44.1) | 47 (42.7) |  |
| In ICU at first positive blood culture | 5 (8.5) | 12 (10.9) | 0.698 |
| CCWI score (median [IQR]) | 5.0 [2.0-7.0] | 5.0 [3.0-7.0] | 0.146 |
| Pitt bacteremia score (median [IQR]) | 1.0 [0.0-3.0] | 1.0 [0.0-3.0] | 0.399 |
| SOFA score (median [IQR]) | 4.0 [1.0-8.0] | 4.0 [2.0-7.0] | 0.863 |
| Severity |  |  | 0.338 |
| Non-sepsis | 9 (15.3) | 21 (20.0) |  |
| sepsis | 35 (59.3) | 50 (47.6) |  |
| severe sepsis | 8 (13.6) | 12 (11.4) |  |
| septic shock | 7 (11.9) | 22 (21.0) |  |
| Primary site of infection |  |  | 0.039 |
| Central venous catheter | 15 (25.4) | 16 (14.9) |  |
| Bone and joint | 14 (23.7) | 10 (9.1) |  |
| Skin and soft tissue | 7 (11.9) | 13 (12.1) |  |
| Lower respiratory tract | 6 (10.2) | 12 (11.1) |  |
| Cardiovascular site^†^ | 9 (15.3) | 18 (16.4) |  |
| Unknown | 6 (10.2) | 28 (25.9) |  |
| Others^‡^ | 2 (3.4) | 11 (10.0) |  |
| Treatment |  |  |  |
| Appropriate empirical | 40 (67.8) | 79 (71.8) | 0.585 |
| Appropriate definitive^§^ | 59 (100) | 93 (87.7) | 0.005 |
| Time until appropriate antibiotics (mean ± SD) (h) | 30.5 ± 26.3 | 25.2 ± 47.6 | 0.435 |
| SAB-related 30-day mortality | 10 (16.9) | 25 (22.7) | 0.007 |
| Persistent SAB | 14 (23.7) | 17 (15.5) | 0.185 |
| Metastatic SAB | 6 (10.2) | 9 (8.6) | 0.733 |

Data are given as number (%), unless otherwise specified.

^†^ includes infective endocarditis and other endovascular infections

^‡^ intraabdominal, urinary tract infection, and central nervous system

^§^ treatment with susceptible antibiotics

MRSAB, methicillin-resistant *S. aureus* bacteremia; IQR, interquartile range; SD, standard deviation; CCWI, Charlson’s comorbidity-weighted index; SOFA, sequential organ failure assessment.

Supplementary Table 2. Relative *TLR2* mRNA expression levels and cytokine concentrations within day 5 of *Staphylococcus aureus* bacteremia (SAB) based on early mortality (mortality within 7 d of SAB onset)

| Variables | Early mortality  (n=3) | Patients surviving the first 7 days* (n=56) | *P* |
| --- | --- | --- | --- |
| Age (mean [range]) (years) | 68.0 [48-82] | 60.3 [26-85] | 0.397 |
| Male | 2 (66.7) | 44 (78.6) | 0.533 |
| MRSAB | 2 (66.7) | 34 (60.7) | 1.00 |
| CCWI (median [range]) | 3 [3-4] | 5 [0-11] | 0.579 |
| Pitt bacteremia score (median [range]) | 4.0 [0.0-10.0] | 1.0 [0.0-5.0] | 0.215 |
| SOFA score (median [range]) | 12.0 [7.0-20.0] | 4.0 [0.0-16.0] | 0.025 |
| Treatment |  |  |  |
| Appropriate empirical | 2 (66.7) | 38 (67.9) | 1.00 |
| Appropriate definitive^§^ | 3 (100) | 56 (100) |  |
| Time until appropriate antibiotics (mean ± SD) (h) | 37.3 ± 17.9 | 30.1 ± 26.7 | 0.407 |
| Persistent SAB | 2 (66.7) | 12 (21.4) | 0.137 |
| Relative TLR2 expression level (median [range]) | 0.14 [0.03-0.22] | 0.26 [0.04-2.29] ^†^ | 0.077 |
| TNF-α (median [range]) (pg/mL) | 11.44 [3.06-28.74] | 5.97 [2.11-34.44] ^‡^ | 0.463 |
| IL-10 concentrations (median [range]) (pg/mL) | 22.46 [8.47-37.94] | 2.09 [0.35-14.25] ^‡^ | 0.006 |
| IL-6 concentrations (median [range]) (pg/mL) | 168.93 [101.11-576.95] | 11.91 [2.51-28.82] ^‡^ | 0.008 |
| IL-10 / TNF-α (median [range]) | 1.32 [0.74-7.33] | 0.28 [0.09-1.39] ^‡^ | 0.014 |

Data are given as number (%), unless otherwise specified.

* n=56 unless otherwise specified

^†^ n=50

^‡^ n=43

MRSAB, methicillin-resistant *S. aureus* bacteremia; CCWI, Charlson’s comorbidity-weighted index; SOFA, sequential organ failure assessment; SD, standard deviation; TNF, tumor necrosis factor; IL, interleukin.

Supplementary Figure 1. Relative *TLR2* mRNA expression levels relative to those of *GAPDH* mRNA among all patients.


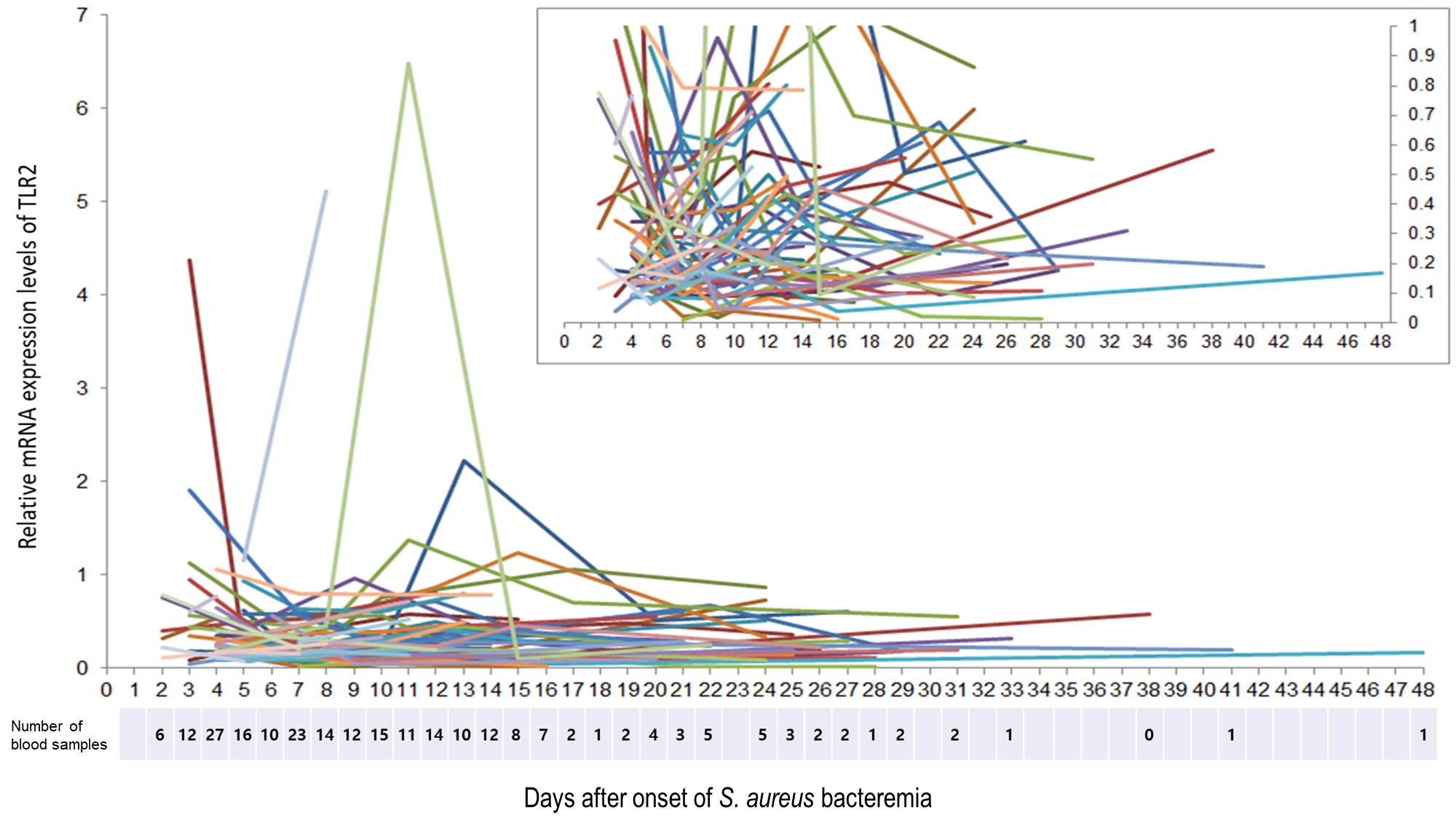


Supplementary Figure 2. Relative *TLR2* mRNA expression levels based on 30-d mortality.
